# Case Report: Delayed Presentation of Bowel Obstruction Caused by Blunt Abdominal Trauma

**DOI:** 10.5811/cpcem.2020.9.47034

**Published:** 2020-10-20

**Authors:** Nicholas H. George, Charles A. Baldi, James M. Tonascia, Siamak Moayedi

**Affiliations:** University of Maryland School of Medicine, Department of Emergency Medicine, Baltimore, Maryland

**Keywords:** Bowel obstruction, blunt trauma, delayed presentation

## Abstract

**Introduction:**

Bowel obstruction is a rare but well reported complication of blunt abdominal trauma (BAT). Obstruction is most often seen acutely caused by bowel wall hematomas and chronically as a result of post-traumatic strictures. Here, we present a novel case of BAT causing a subacute obstructing bowel wall hematoma.

**Case Report:**

A healthy, 32-year-old male presented to our emergency department with three days of nausea and vomiting. Chart review revealed he had been seen two weeks prior after a high-speed motor vehicle collision. During that initial visit, the patient had a benign abdominal exam and was discharged without imaging. On this return visit, the patient was found to have a large, obstructing colonic hematoma.

**Conclusion:**

Because emergency physicians care for patients in both the acute and subacute phases of trauma, clinicians should recognize the more subtle sequelae of BAT.

## INTRODUCTION

Bowel obstruction is a common diagnosis made in the emergency department (ED). Classic causes include strictures, hernias, and malignancy. Rarely, clinicians may see a wide range of less common precipitants, from foreign objects to enteroliths.^1^ Abdominal trauma is one appreciated cause of bowel obstruction. In the acute setting, blunt trauma can lead to rapidly expanding bowel wall hematomas.^2^ Such injury complexes are often rapidly identified on imaging, and managed with emergency surgery. In the delayed setting, blunt trauma can cause adhesions that might secondarily lead to bowel obstruction.^3^ Here we present a novel case of a subacute traumatic bowel wall hematoma causing intestinal obstruction. To our knowledge, delayed intestinal obstruction caused by traumatic bowel wall hematoma has not been reported previously.

## CASE REPORT

A 32-year-old male presented to the ED after experiencing three days of nausea and vomiting. Additionally, he reported anorexia with constipation for three days, and obstipation for one day. He had no relevant past medical history, was taking no medications, and had no allergies. Review of the electronic health record revealed the patient had been evaluated two weeks prior following a motor vehicle collision (MVC). He had been the restrained driver in a two-car collision with associated airbag deployment and heavy front-end damage. He was seen approximately 30 minutes after the MVC at a community hospital without a trauma center designation. At the time, he was hemodynamically stable and did not have any abdominal pain or bruising. He was evaluated by an attending physician and, after a primary and secondary trauma survey, was discharged without any indication for imaging or further evaluation.

For the current ED visit, his presenting vital signs were as follows: temperature 97.8 degrees Fahrenheit; heart rate 89 beats per minute; blood pressure 126/73 millimeters of mercury (mm Hg); respiratory rate 20 breaths per minute; and oxygen saturation 98% on room air. Physical examination revealed a soft but diffusely tender abdomen with voluntary guarding in all quadrants. There was no rebound, rigidity, or palpable mass. Further, no external signs of trauma were appreciated. The remainder of his examination was unremarkable, including bowel sounds, which were present and normoactive. On history-taking he denied new trauma aside from the known MVC, and had been taking acetaminophen since the accident for pain control.

Notable laboratory values were as follows: hemoglobin 13.2 grams (g) per deciliter (dL) (reference [ref] range: 13.5–16.5 g/dL), hematocrit 40.4 % (40.0–49.0% ref range), anion gap 9 milliequivalents (mEq) per liter (L) (ref range: 5–17 mEq/L), aspartate aminotransferase 21 international units (IU) per L (ref range: 8–40 IU/L), and alanine aminotransferase 14 IU/L (ref range: 15–45 IU/L). While awaiting computed tomography (CT), a point-of-care abdominal ultrasound examination was completed. There was no free fluid visualized in the hepatorenal, splenorenal, or pelvic windows. Further, there was no mass visualized within the abdominal cavity. Subsequently, a CT of the abdomen with intravenous (IV) contrast revealed a large intramural fluid collection occluding the lumen of the ascending colon, with resulting bowel obstruction. (Image).

We inserted a nasogastric tube – with return of non-bilious stomach contents – for gastric decompression and administered IV fluids. He was admitted to the surgical service for observation and was taken to the operating room two days later for bowel resection. He was discharged on postoperative day six. His course was complicated by a minor surgical site infection, which was managed with point-of-care drainage. No antibiotics were required. At his five-week follow-up appointment, he reported normal bowel movements and eating habits, and was cleared to resume full activity.

## DISCUSSION

Causes of post-traumatic bowel obstruction include hematoma formation, bowel wall edema, and late-presenting strictures.^1^,^2^, ^3^ Complications from hematomas or bowel wall edema typically present within hours to days. These injuries – such as bucket-handle mesenteric injuries, ruptured viscera, and mesenteric lacerations – are often detectable with CT at the time of presentation.^4^ Such cases are often described in the duodenum and are mostly self-limited.^5^ Strictures, on the other hand, commonly present in a more delayed fashion between one month and two years after BAT. One case report detailed a patient with traumatic adhesions causing small bowel obstruction more than 20 years after the incident.^6^ Our case does not follow this paradigm and is unique in both location and timing. Our patient’s obstructing hematoma was in the ascending colon as opposed to the duodenum, and he became symptomatic two weeks after the initial injury.

CPC-EM Capsule

What do we already know about this clinical entity?

*Post-traumatic bowel obstructions are known to occur within hours after trauma due to hematoma formation. Late-presenting obstructions are often due to strictures*.

What makes this presentation of disease reportable?

*A late-presenting, sub-acute bowel wall hematoma causing a post-traumatic obstruction has not been previously described in the literature*.

What is the major learning point?

*Intra-abdominal injury in the form of bowel obstruction from blunt abdominal trauma may only become apparent weeks after the incident*.

How might this improve emergency medicine practice?

*Patients presenting with abdominal complaints in the post-trauma setting should be evaluated for intra-abdominal pathology despite a previously negative exam*.

Cross-sectional imaging is crucial to the diagnosis and treatment of these injuries. The best imaging modality to diagnose bowel obstruction is CT with IV and oral contrast. Unfortunately, the patient in this case was unable to tolerate oral contrast due to unremitting nausea and vomiting. While the dual-contrast approach would have allowed us to better visualize smaller, more nuanced causes of bowel obstruction, we found an IV-contrast only approach was sufficient for this case.^7^

Within this context, it is notable that CT with IV contrast is preferable to ultrasound (US). In this case, point-of-care US was attempted but was not diagnostic. Ultrasound has been described as an alternative imaging technique for diagnosing intestinal obstruction, appendicitis, and other bowel diseases.^8^ However, as our case illustrates, US is unlikely to identify the etiology and location of a post-traumatic obstruction. The role of US in delayed presentation after blunt abdominal trauma may be limited to simply identifying the presence of a bowel obstruction or abdominal free fluid.

As the management of these conditions is nuanced and complex, it is imperative they are diagnosed in a timely manner. Yet subacute and rare presentations, like this one, risk going unnoticed. Based on our patient’s initial presentation to the ED, directly after his trauma, clinical guidelines did not point toward the need for additional workup.^8^ If abdominal CT imaging had been used at that visit, right after his injury, it is unclear whether any pathology would have been identified. In this way, emergency physicians should be aware that a patient with recent trauma and a negative CT (or no indication for imaging at all) might return to the ED with a related, clinically significant condition.

Initial management of patients with intraluminal hematoma includes bowel rest, insertion of a nasogastric tube for intestinal decompression, and observation. Definitive management is more intricate. Hematomas can be resorbed, some rapidly and spontaneously, but others have taken months.^9^ If a mechanical obstruction is identified, patients should be admitted to a surgical service for management and potential operative intervention, as was done in this case.

## CONCLUSION

As this case demonstrates, emergency physicians should consider complications from recent trauma even in the absence of indications for imaging on initial presentation. A patient who presents with symptoms of bowel obstruction after sustaining remote blunt trauma should undergo a CT of the abdomen. As EDs often care for patients both in the immediate aftermath of traumatic injury and later in their lives, emergency physicians must recognize the more subtle, subacute effects of trauma, in this case, blunt abdominal trauma.

## Figures and Tables

**Image:**
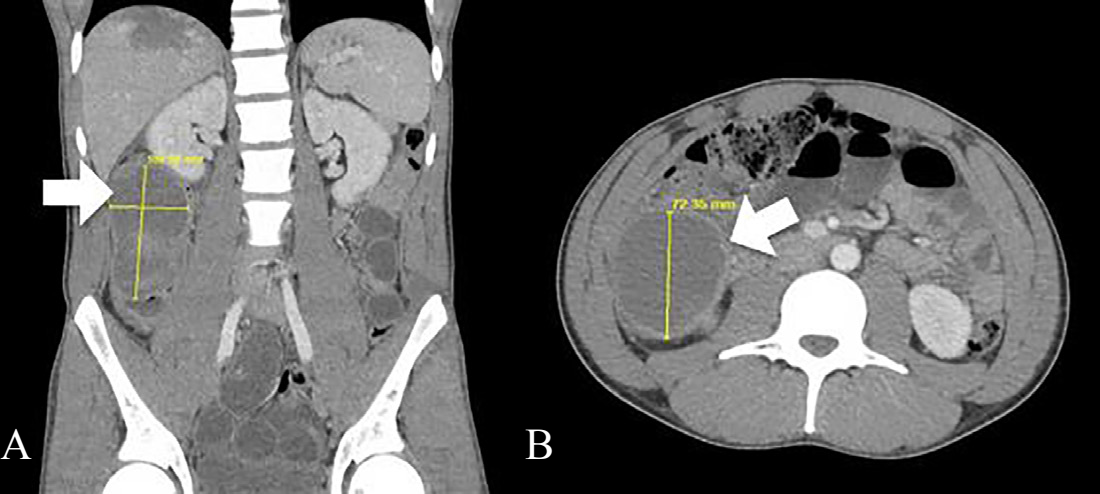
Computed tomography with contrast, showing a large (10.1×5.9×7.2 centimeters), heterogeneous intraluminal collection of fluid in the ascending colon (arrows). A. Coronal view. B. Axial view.
